# How can key findings from patients with Urbach-Wiethe Disease (UWD) support the role of amygdala in socio-emotional-cognitive functioning? The case of a young adult with genetically proven UWD without amygdala calcifications

**DOI:** 10.1192/j.eurpsy.2023.1295

**Published:** 2023-07-19

**Authors:** A. Staniloiu, H. J. Markowitsch

**Affiliations:** 1 Psychology, University of Bielefeld, Bielefeld; 2 Psychology, University of Bielefeld, Bielefeld, Germany

## Abstract

**Introduction:**

Urbach-Wiethe disease (UWD; also named *Lipoid proteinosis* or *Hyalinosis cutis et mucosae*) was first described in 1929 by the Austrian scientists Erich Urbach and Camillo Wiethe and constitutes an autosomal recessive disorder which is characterized by several changes of body and brain. Most patients – and especially older ones – show symmetrical calcifications in the medial temporal lobes, especially the amygdala and the periamygdaloid region (Siebert *et al*., Brain 2003, 126, 2627-2637).

Patients with UWD with bilateral amygdala calcifications show several changes, from impairments in interpreting of odors to more complex changes in socio-cognitive and emotional domains (Markowitsch & Staniloiu, *Neuropsychologia*,2011. 49, 718-733).

**Objectives:**

Here, we describe the rarer case of a 19-year-old man with genetically proven UWD, who – up to now – lacks significant brain calcification.

**Methods:**

The patient was investigated medically, psychiatrically and with neuropsychological and neuroimaging methods.

**Results:**

Findings of CT (see Figure 1) and MRI scans yielded no evidence of significant brain calcifications. Our patient AC manifested only a subset of changes encountered in patients with UWD with bilateral amygdala calcifications, namely in emotional processing (such as in more complex subsets of the Florida Affect Battery and Recall of Emotional/Neutral photographs), social cognition (Reading the Mind in the Eyes) and personality dimensions (suggestions for obsessive tendencies). The impairments in emotion-related task performance were similar in extent to those of the three UWD patients with bilateral amygdala calcifications of Brand *et al.*. Neuropsychologia 2007, *45*, 1305-1317., indicating a probable sub-normal amygdalar functioning, in the absence of evidence of macrostructural amygdalar changes on imaging.

In the Game-of-Dice Task 17 out of 18 trials were so-called safe trials. His intelligence quotient (IQ) was 124 (33 points; MWWT-B), likely being a prerequisite for managing the requirements of the Game-of-Dice Task, enabling him to suppress impulsive acts.

**Conclusions:**

Calcifications in Urbach-Wiethe disease take place progressively- possibly underpinned by genetic and gender variables; this can subsequently allow psychosocial-social factors (such as education and socialization) and biological factors (compensatory neuroplasticity) to retard and diminish the development of socio-emotional and cognitive deteriorations,

Given that select lesions to the human amygdala are exceedingly rare, longitudinal studies of patients with the UWD provide key evidence about how slowly progressive, developmental changes of the amygdala modulate vulnerability to socio-cognitive-emotional impairments and psychopathology.

**Disclosure of Interest:**

None Declared

